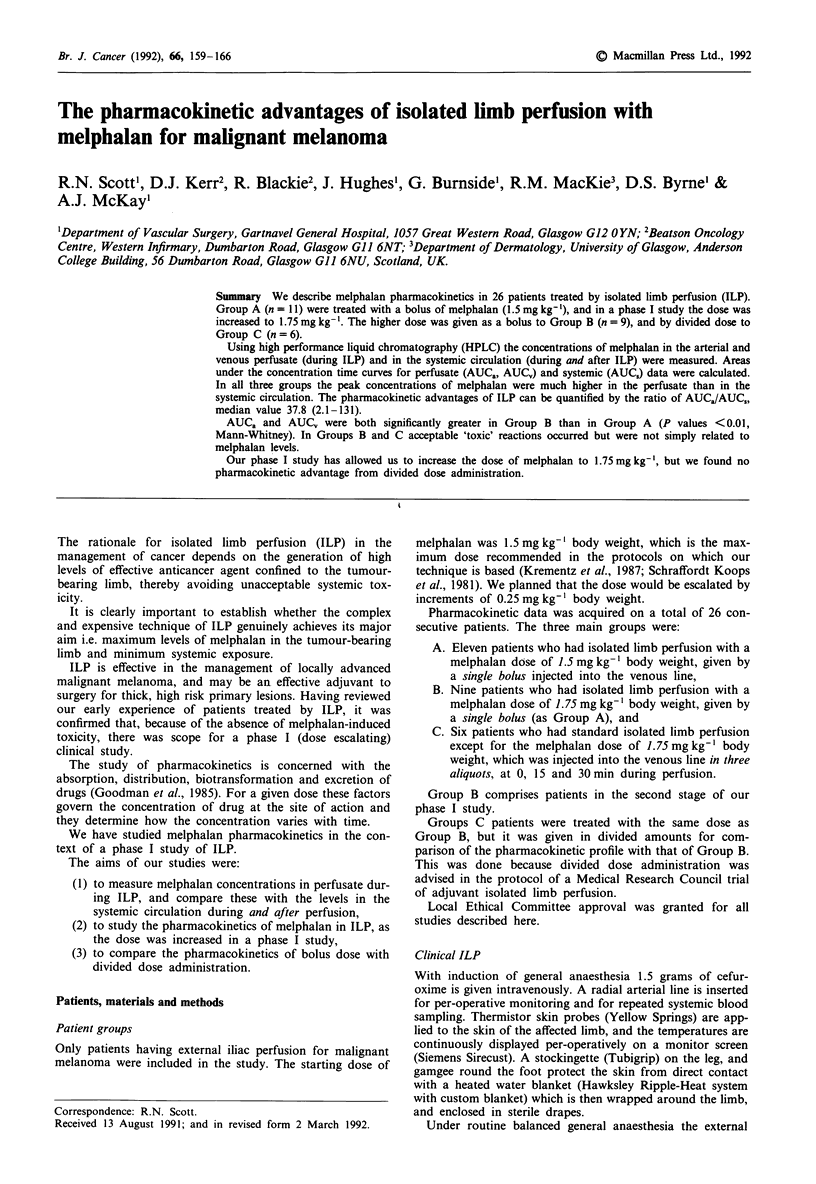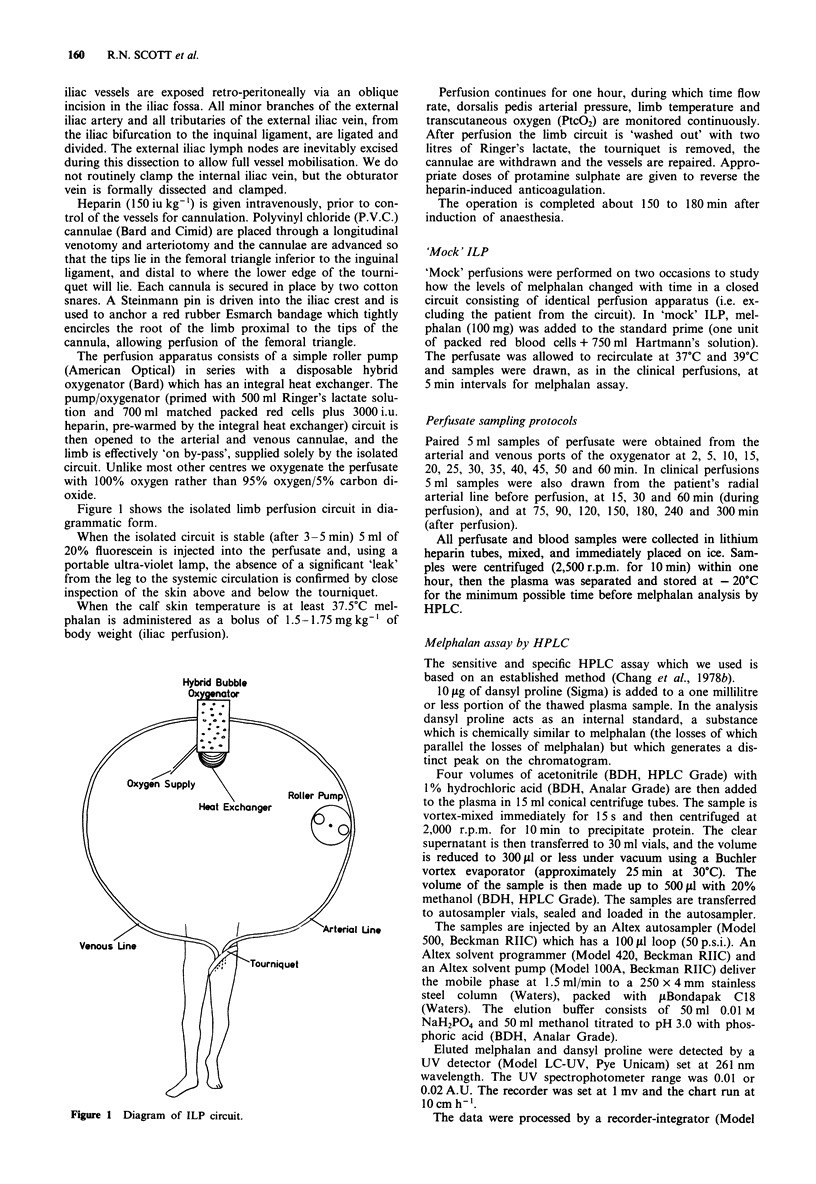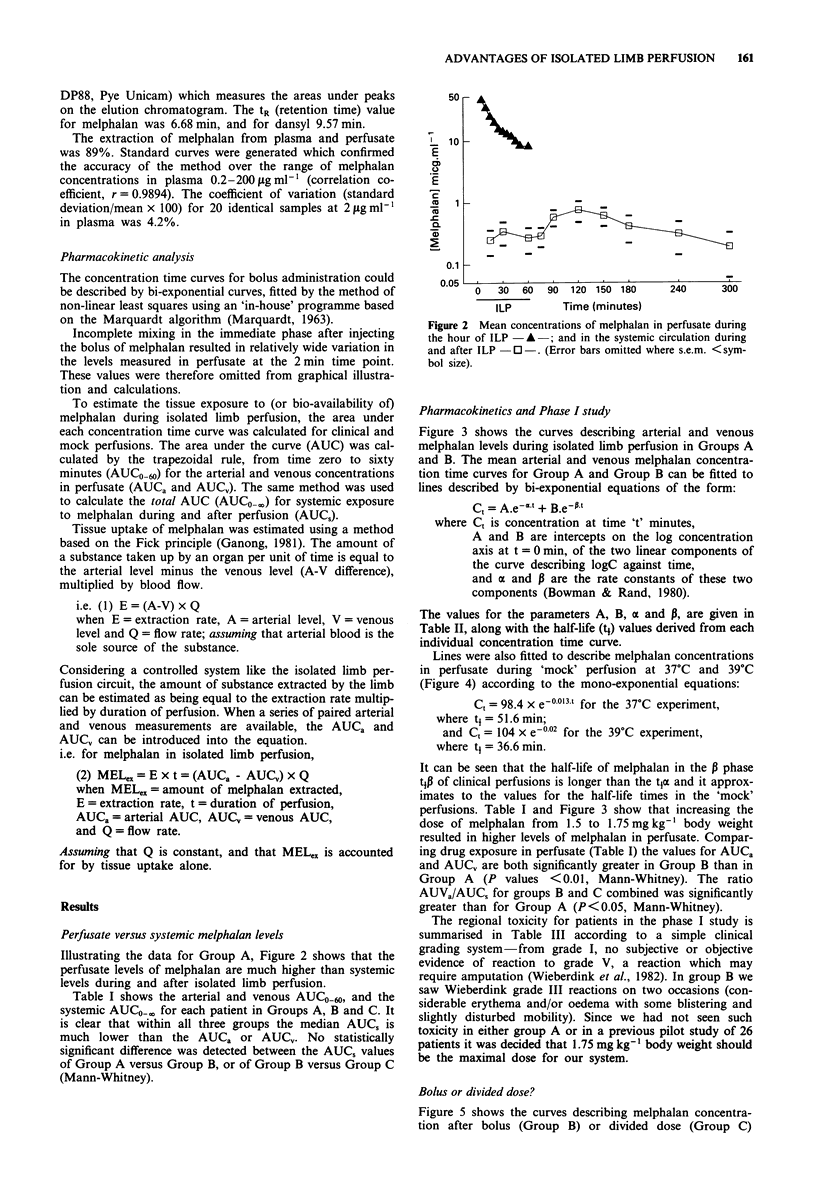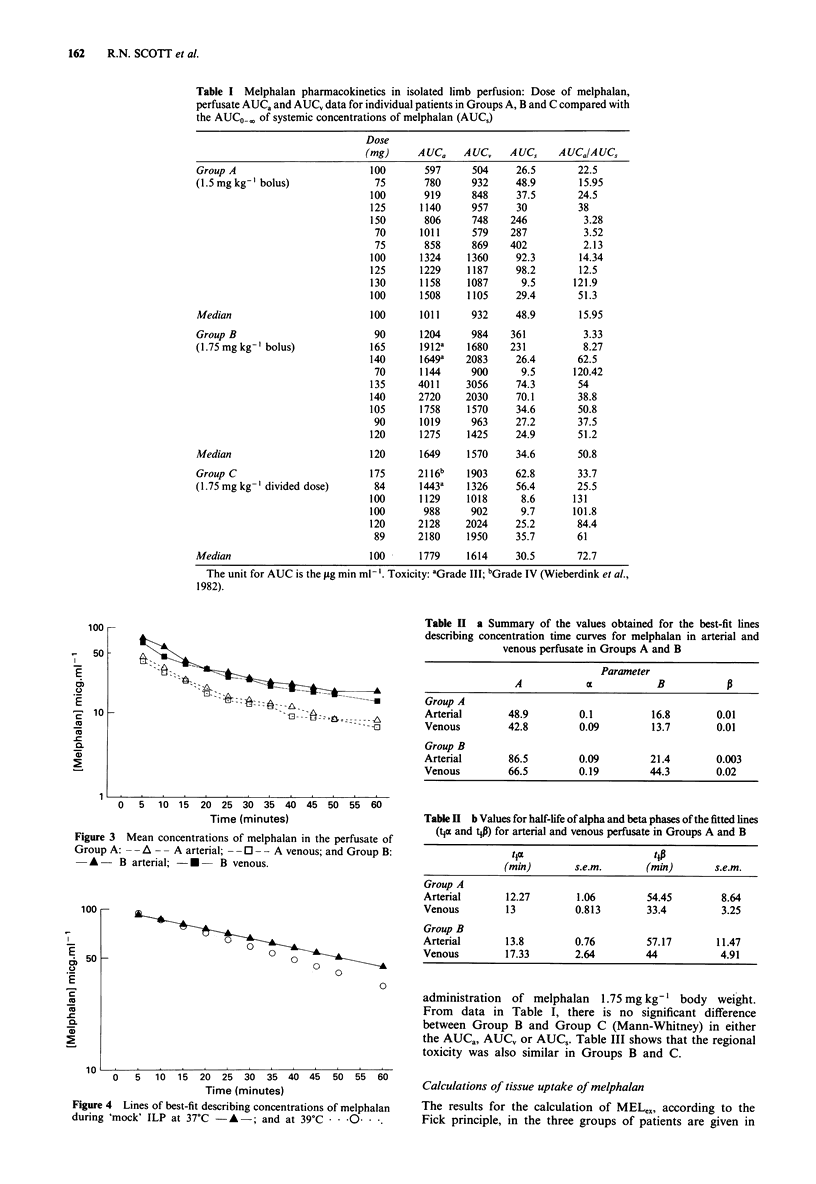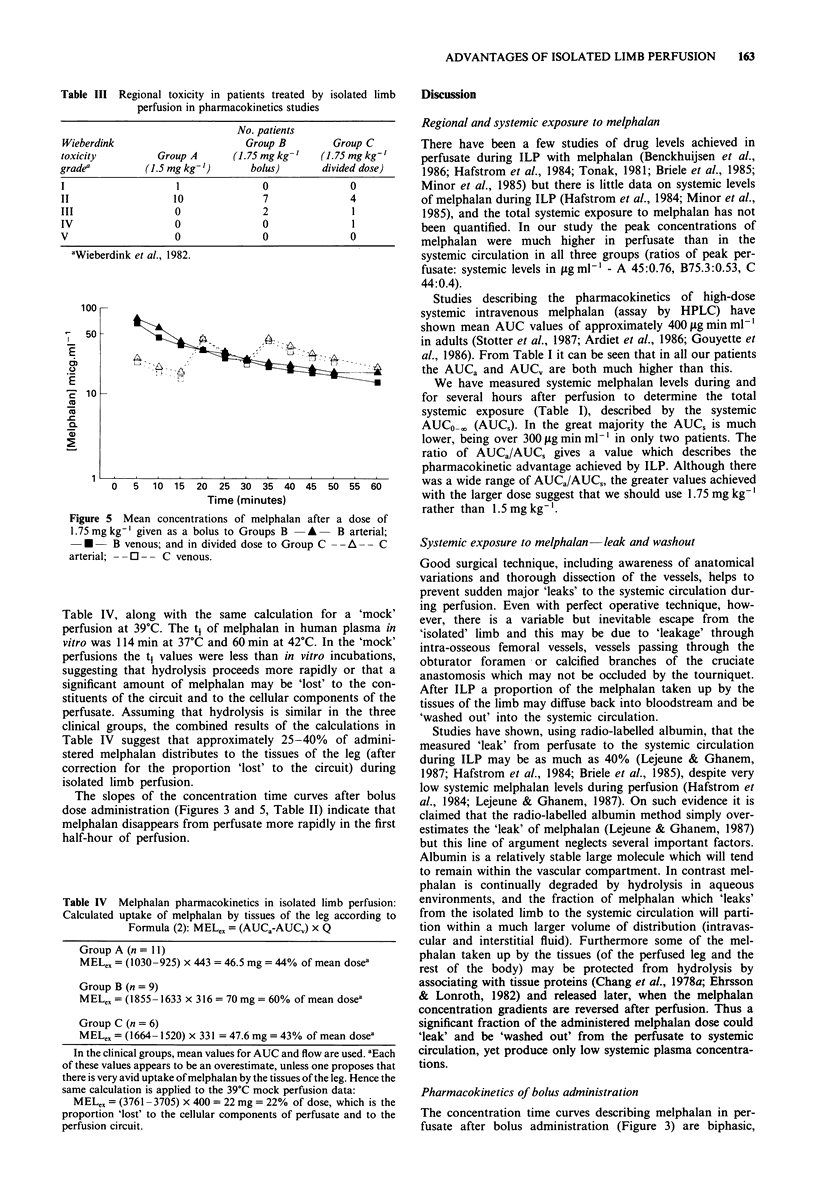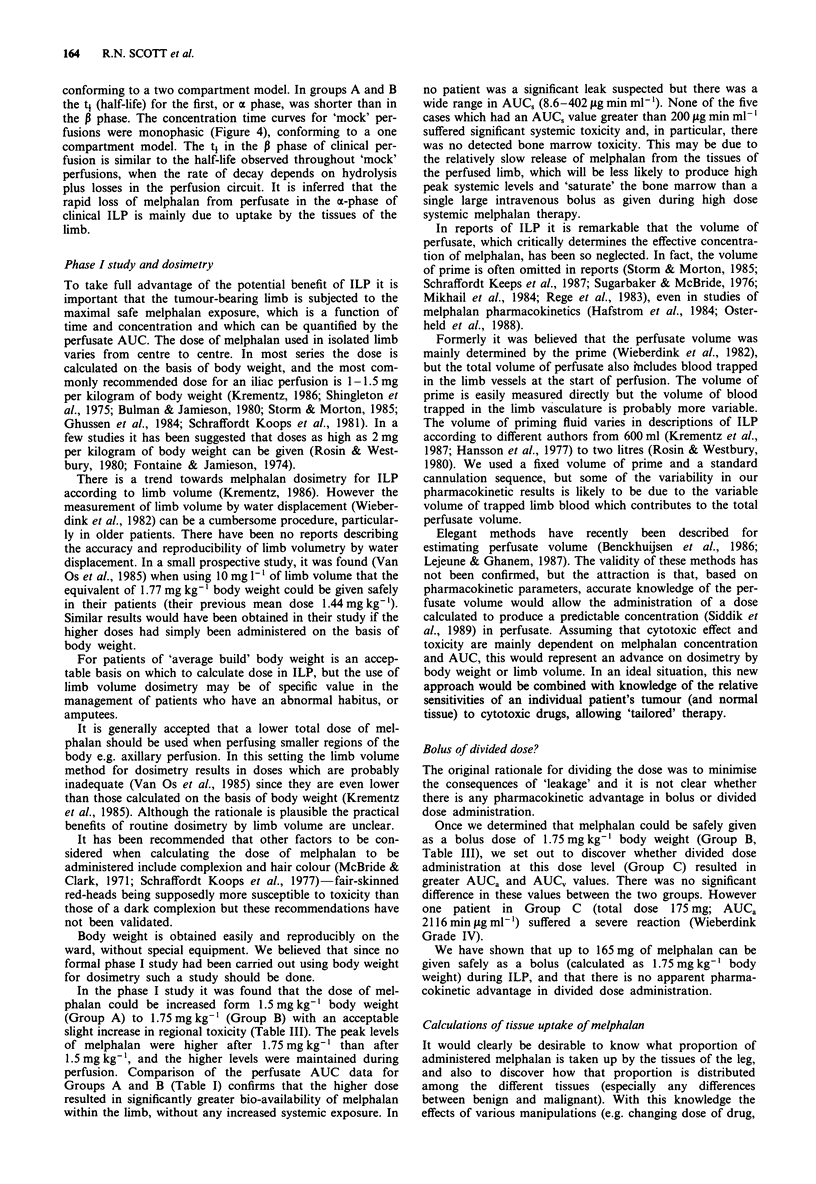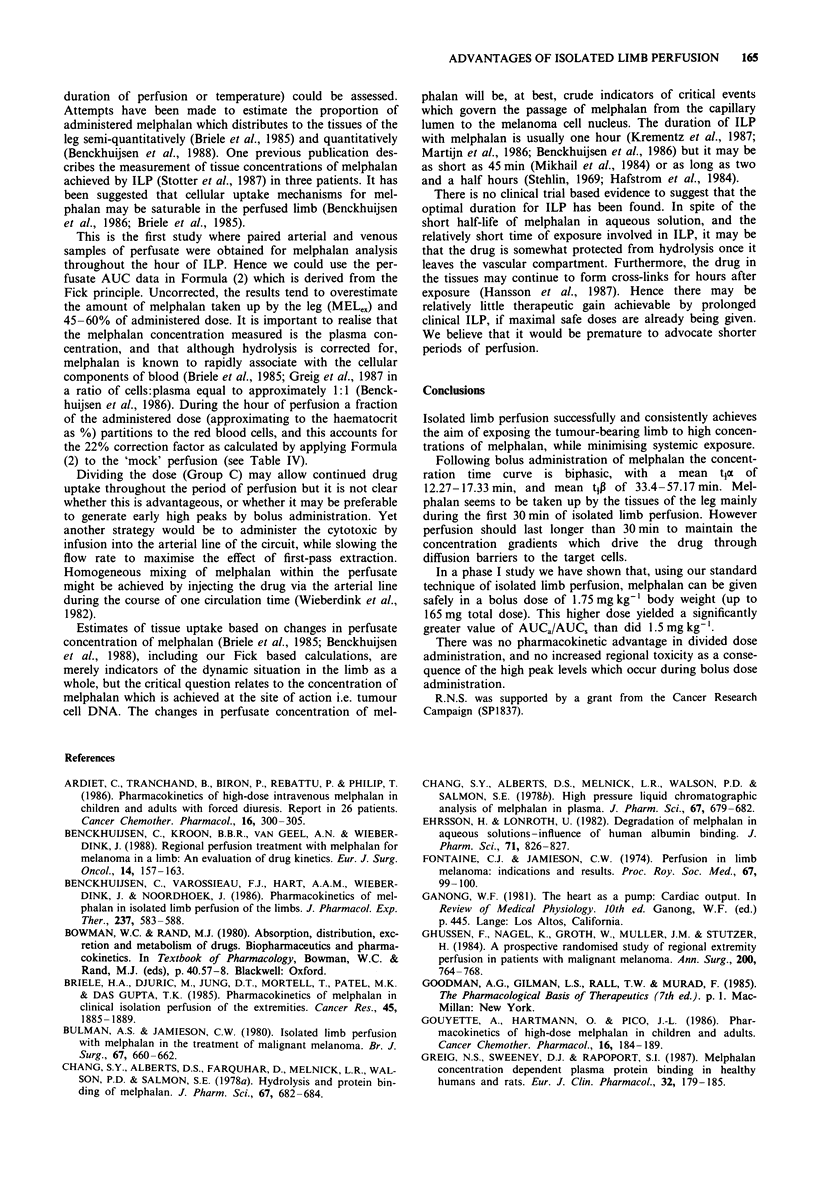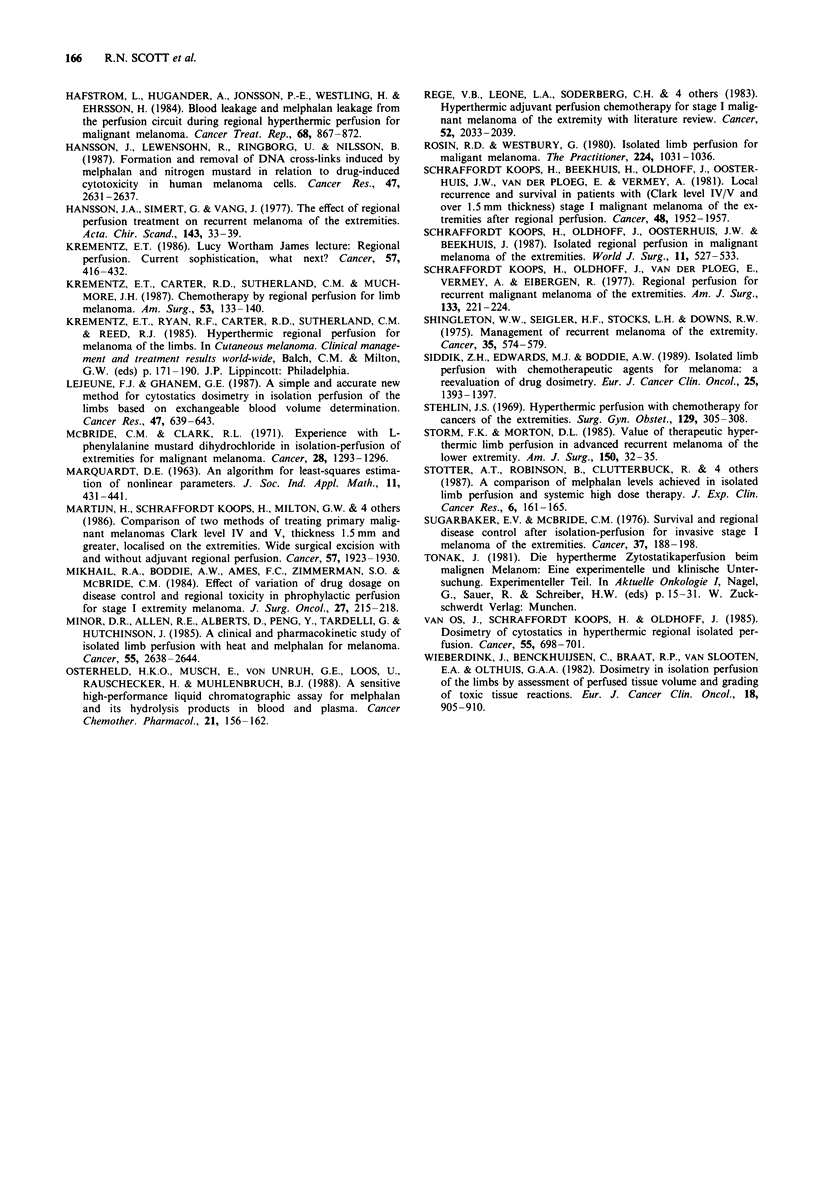# The pharmacokinetic advantages of isolated limb perfusion with melphalan for malignant melanoma.

**DOI:** 10.1038/bjc.1992.235

**Published:** 1992-07

**Authors:** R. N. Scott, D. J. Kerr, R. Blackie, J. Hughes, G. Burnside, R. M. MacKie, D. S. Byrne, A. J. McKay

**Affiliations:** Department of Vascular Surgery, Gartnavel General Hospital, Glasgow, Scotland, UK.

## Abstract

We describe melphalan pharmacokinetics in 26 patients treated by isolated limb perfusion (ILP). Group A (n = 11) were treated with a bolus of melphalan (1.5 mg kg-1), and in a phase I study the dose was increased to 1.75 mg kg-1. The higher dose was given as a bolus to Group B (n = 9), and by divided dose to Group C (n = 6). Using high performance liquid chromatography (HPLC) the concentrations of melphalan in the arterial and venous perfusate (during ILP) and in the systemic circulation (during and after ILP) were measured. Areas under the concentration time curves for perfusate (AUCa, AUCv) and systemic (AUCs) data were calculated. In all three groups the peak concentrations of melphalan were much higher in the perfusate than in the systemic circulation. The pharmacokinetic advantages of ILP can be quantified by the ratio of AUCa/AUCs, median value 37.8 (2.1-131). AUCa and AUCv were both significantly greater in Group B than in Group A (P values less than 0.01, Mann-Whitney). In Groups B and C acceptable 'toxic' reactions occurred but were not simply related to melphalan levels. Our phase I study has allowed us to increase the dose of melphalan to 1.75 mg kg-1, but we found no pharmacokinetic advantage from divided dose administration.


*Br. J. Cancer* (1992), **66**, 159–166 © Macmillan Press Ltd., 1992

### The pharmacokinetic advantages of isolated limb perfusion with melphalan for malignant melanoma

R.N. Scott[1], D.J. Kerr[2], R. Blackie[2], J. Hughes[1], G. Burnside[1], R.M. MacKie[3], D.S. Byrne[1] & A.J. McKay[1]

[1]*Department of Vascular Surgery, Gartnavel General Hospital, 1057 Great Western Road, Glasgow G12 0YN;* [2]*Beatson Oncology Centre, Western Infirmary, Dumbarton Road, Glasgow G11 6NT;* [3]*Department of Dermatology, University of Glasgow, Anderson College Building, 56 Dumbarton Road, Glasgow G11 6NU, Scotland, UK.*

**Summary** We describe melphalan pharmacokinetics in 26 patients treated by isolated limb perfusion (ILP). Group A ($n = 11$) were treated with a bolus of melphalan (1.5 mg kg$^{-1}$), and in a phase I study the dose was increased to 1.75 mg kg$^{-1}$. The higher dose was given as a bolus to Group B ($n = 9$), and by divided dose to Group C ($n = 6$).

Using high performance liquid chromatography (HPLC) the concentrations of melphalan in the arterial and venous perfusate (during ILP) and in the systemic circulation (during *and* after ILP) were measured. Areas under the concentration time curves for perfusate ($AUC_a$, $AUC_v$) and systemic ($AUC_s$) data were calculated. In all three groups the peak concentrations of melphalan were much higher in the perfusate than in the systemic circulation. The pharmacokinetic advantages of ILP can be quantified by the ratio of $AUC_a/AUC_s$, median value 37.8 (2.1–131).

$AUC_a$ and $AUC_v$ were both significantly greater in Group B than in Group A ($P$ values $<0.01$, Mann-Whitney). In Groups B and C acceptable 'toxic' reactions occurred but were not simply related to melphalan levels.

Our phase I study has allowed us to increase the dose of melphalan to 1.75 mg kg$^{-1}$, but we found no pharmacokinetic advantage from divided dose administration.

The rationale for isolated limb perfusion (ILP) in the management of cancer depends on the generation of high levels of effective anticancer agent confined to the tumour-bearing limb, thereby avoiding unacceptable systemic toxicity.

It is clearly important to establish whether the complex and expensive technique of ILP genuinely achieves its major aim i.e. maximum levels of melphalan in the tumour-bearing limb and minimum systemic exposure.

ILP is effective in the management of locally advanced malignant melanoma, and may be an effective adjuvant to surgery for thick, high risk primary lesions. Having reviewed our early experience of patients treated by ILP, it was confirmed that, because of the absence of melphalan-induced toxicity, there was scope for a phase I (dose escalating) clinical study.

The study of pharmacokinetics is concerned with the absorption, distribution, biotransformation and excretion of drugs (Goodman *et al.*, 1985). For a given dose these factors govern the concentration of drug at the site of action and they determine how the concentration varies with time.

We have studied melphalan pharmacokinetics in the context of a phase I study of ILP.

The aims of our studies were:

(1) to measure melphalan concentrations in perfusate during ILP, and compare these with the levels in the systemic circulation during *and after* perfusion,
(2) to study the pharmacokinetics of melphalan in ILP, as the dose was increased in a phase I study,
(3) to compare the pharmacokinetics of bolus dose with divided dose administration.

### Patients, materials and methods

### *Patient groups*

Only patients having external iliac perfusion for malignant melanoma were included in the study. The starting dose of melphalan was 1.5 mg kg$^{-1}$ body weight, which is the maximum dose recommended in the protocols on which our technique is based (Krementz *et al.*, 1987; Schraffordt Koops *et al.*, 1981). We planned that the dose would be escalated by increments of 0.25 mg kg$^{-1}$ body weight.

Pharmacokinetic data was acquired on a total of 26 consecutive patients. The three main groups were:

A. Eleven patients who had isolated limb perfusion with a melphalan dose of *1.5* mg kg$^{-1}$ body weight, given by a *single bolus* injected into the venous line,
B. Nine patients who had isolated limb perfusion with a melphalan dose of *1.75* mg kg$^{-1}$ body weight, given by a *single bolus* (as Group A), and
C. Six patients who had standard isolated limb perfusion except for the melphalan dose of *1.75* mg kg$^{-1}$ body weight, which was injected into the venous line *in three aliquots*, at 0, 15 and 30 min during perfusion.

Group B comprises patients in the second stage of our phase I study.

Groups C patients were treated with the same dose as Group B, but it was given in divided amounts for comparison of the pharmacokinetic profile with that of Group B. This was done because divided dose administration was advised in the protocol of a Medical Research Council trial of adjuvant isolated limb perfusion.

Local Ethical Committee approval was granted for all studies described here.

### *Clinical ILP*

With induction of general anaesthesia 1.5 grams of cefuroxime is given intravenously. A radial arterial line is inserted for per-operative monitoring and for repeated systemic blood sampling. Thermistor skin probes (Yellow Springs) are applied to the skin of the affected limb, and the temperatures are continuously displayed per-operatively on a monitor screen (Siemens Sirecust). A stockingette (Tubigrip) on the leg, and gamgee round the foot protect the skin from direct contact with a heated water blanket (Hawksley Ripple-Heat system with custom blanket) which is then wrapped around the limb, and enclosed in sterile drapes.

Under routine balanced general anaesthesia the external

Correspondence: R.N. Scott.
Received 13 August 1991; and in revised form 2 March 1992.

iliac vessels are exposed retro-peritoneally via an oblique incision in the iliac fossa. All minor branches of the external iliac artery and all tributaries of the external iliac vein, from the iliac bifurcation to the inquinal ligament, are ligated and divided. The external iliac lymph nodes are inevitably excised during this dissection to allow full vessel mobilisation. We do not routinely clamp the internal iliac vein, but the obturator vein is formally dissected and clamped.

Heparin (150 iu $kg^{-1}$) is given intravenously, prior to control of the vessels for cannulation. Polyvinyl chloride (P.V.C.) cannulae (Bard and Cimid) are placed through a longitudinal venotomy and arteriotomy and the cannulae are advanced so that the tips lie in the femoral triangle inferior to the inguinal ligament, and distal to where the lower edge of the tourniquet will lie. Each cannula is secured in place by two cotton snares. A Steinmann pin is driven into the iliac crest and is used to anchor a red rubber Esmarch bandage which tightly encircles the root of the limb proximal to the tips of the cannula, allowing perfusion of the femoral triangle.

The perfusion apparatus consists of a simple roller pump (American Optical) in series with a disposable hybrid oxygenator (Bard) which has an integral heat exchanger. The pump/oxygenator (primed with 500 ml Ringer's lactate solution and 700 ml matched packed red cells plus 3000 i.u. heparin, pre-warmed by the integral heat exchanger) circuit is then opened to the arterial and venous cannulae, and the limb is effectively 'on by-pass', supplied solely by the isolated circuit. Unlike most other centres we oxygenate the perfusate with 100% oxygen rather than 95% oxygen/5% carbon dioxide.

Figure 1 shows the isolated limb perfusion circuit in diagrammatic form.

When the isolated circuit is stable (after 3–5 min) 5 ml of 20% fluorescein is injected into the perfusate and, using a portable ultra-violet lamp, the absence of a significant 'leak' from the leg to the systemic circulation is confirmed by close inspection of the skin above and below the tourniquet.

When the calf skin temperature is at least 37.5°C melphalan is administered as a bolus of 1.5–1.75 mg $kg^{-1}$ of body weight (iliac perfusion).

**Figure 1** Diagram of ILP circuit.

Perfusion continues for one hour, during which time flow rate, dorsalis pedis arterial pressure, limb temperature and transcutaneous oxygen ($PtcO_2$) are monitored continuously. After perfusion the limb circuit is 'washed out' with two litres of Ringer's lactate, the tourniquet is removed, the cannulae are withdrawn and the vessels are repaired. Appropriate doses of protamine sulphate are given to reverse the heparin-induced anticoagulation.

The operation is completed about 150 to 180 min after induction of anaesthesia.

### *'Mock' ILP*

'Mock' perfusions were performed on two occasions to study how the levels of melphalan changed with time in a closed circuit consisting of identical perfusion apparatus (i.e. excluding the patient from the circuit). In 'mock' ILP, melphalan (100 mg) was added to the standard prime (one unit of packed red blood cells + 750 ml Hartmann's solution). The perfusate was allowed to recirculate at 37°C and 39°C and samples were drawn, as in the clinical perfusions, at 5 min intervals for melphalan assay.

### *Perfusate sampling protocols*

Paired 5 ml samples of perfusate were obtained from the arterial and venous ports of the oxygenator at 2, 5, 10, 15, 20, 25, 30, 35, 40, 45, 50 and 60 min. In clinical perfusions 5 ml samples were also drawn from the patient's radial arterial line before perfusion, at 15, 30 and 60 min (during perfusion), and at 75, 90, 120, 150, 180, 240 and 300 min (after perfusion).

All perfusate and blood samples were collected in lithium heparin tubes, mixed, and immediately placed on ice. Samples were centrifuged (2,500 r.p.m. for 10 min) within one hour, then the plasma was separated and stored at − 20°C for the minimum possible time before melphalan analysis by HPLC.

### *Melphalan assay by HPLC*

The sensitive and specific HPLC assay which we used is based on an established method (Chang *et al.*, 1978*b*).

10 μg of dansyl proline (Sigma) is added to a one millilitre or less portion of the thawed plasma sample. In the analysis dansyl proline acts as an internal standard, a substance which is chemically similar to melphalan (the losses of which parallel the losses of melphalan) but which generates a distinct peak on the chromatogram.

Four volumes of acetonitrile (BDH, HPLC Grade) with 1% hydrochloric acid (BDH, Analar Grade) are then added to the plasma in 15 ml conical centrifuge tubes. The sample is vortex-mixed immediately for 15 s and then centrifuged at 2,000 r.p.m. for 10 min to precipitate protein. The clear supernatant is then transferred to 30 ml vials, and the volume is reduced to 300 μl or less under vacuum using a Buchler vortex evaporator (approximately 25 min at 30°C). The volume of the sample is then made up to 500 μl with 20% methanol (BDH, HPLC Grade). The samples are transferred to autosampler vials, sealed and loaded in the autosampler.

The samples are injected by an Altex autosampler (Model 500, Beckman RIIC) which has a 100 μl loop (50 p.s.i.). An Altex solvent programmer (Model 420, Beckman RIIC) and an Altex solvent pump (Model 100A, Beckman RIIC) deliver the mobile phase at 1.5 ml/min to a 250 × 4 mm stainless steel column (Waters), packed with μBondapak C18 (Waters). The elution buffer consists of 50 ml 0.01 M $NaH_2PO_4$ and 50 ml methanol titrated to pH 3.0 with phosphoric acid (BDH, Analar Grade).

Eluted melphalan and dansyl proline were detected by a UV detector (Model LC-UV, Pye Unicam) set at 261 nm wavelength. The UV spectrophotometer range was 0.01 or 0.02 A.U. The recorder was set at 1 mv and the chart run at 10 cm $h^{-1}$.

The data were processed by a recorder-integrator (Model

DP88, Pye Unicam) which measures the areas under peaks on the elution chromatogram. The $t_R$ (retention time) value for melphalan was 6.68 min, and for dansyl 9.57 min.

The extraction of melphalan from plasma and perfusate was 89%. Standard curves were generated which confirmed the accuracy of the method over the range of melphalan concentrations in plasma 0.2–200 $\mu g\, ml^{-1}$ (correlation coefficient, $r = 0.9894$). The coefficient of variation (standard deviation/mean × 100) for 20 identical samples at 2 $\mu g\, ml^{-1}$ in plasma was 4.2%.

*Pharmacokinetic analysis*

The concentration time curves for bolus administration could be described by bi-exponential curves, fitted by the method of non-linear least squares using an 'in-house' programme based on the Marquardt algorithm (Marquardt, 1963).

Incomplete mixing in the immediate phase after injecting the bolus of melphalan resulted in relatively wide variation in the levels measured in perfusate at the 2 min time point. These values were therefore omitted from graphical illustration and calculations.

To estimate the tissue exposure to (or bio-availability of) melphalan during isolated limb perfusion, the area under each concentration time curve was calculated for clinical and mock perfusions. The area under the curve (AUC) was calculated by the trapezoidal rule, from time zero to sixty minutes ($AUC_{0-60}$) for the arterial and venous concentrations in perfusate ($AUC_a$ and $AUC_v$). The same method was used to calculate the *total* AUC ($AUC_{0-\infty}$) for systemic exposure to melphalan during and after perfusion ($AUC_s$).

Tissue uptake of melphalan was estimated using a method based on the Fick principle (Ganong, 1981). The amount of a substance taken up by an organ per unit of time is equal to the arterial level minus the venous level (A-V difference), multiplied by blood flow.

i.e. (1) $E = (A\text{-}V) \times Q$
when E = extraction rate, A = arterial level, V = venous level and Q = flow rate; *assuming* that arterial blood is the sole source of the substance.

Considering a controlled system like the isolated limb perfusion circuit, the amount of substance extracted by the limb can be estimated as being equal to the extraction rate multiplied by duration of perfusion. When a series of paired arterial and venous measurements are available, the $AUC_a$ and $AUC_v$ can be introduced into the equation.
i.e. for melphalan in isolated limb perfusion,

(2) $MEL_{ex} = E \times t = (AUC_a - AUC_v) \times Q$
when $MEL_{ex}$ = amount of melphalan extracted,
E = extraction rate, t = duration of perfusion,
$AUC_a$ = arterial AUC, $AUC_v$ = venous AUC,
and Q = flow rate.

*Assuming* that Q is constant, and that $MEL_{ex}$ is accounted for by tissue uptake alone.

### Results

*Perfusate versus systemic melphalan levels*

Illustrating the data for Group A, Figure 2 shows that the perfusate levels of melphalan are much higher than systemic levels during and after isolated limb perfusion.

Table I shows the arterial and venous $AUC_{0-60}$, and the systemic $AUC_{0-\infty}$ for each patient in Groups A, B and C. It is clear that within all three groups the median $AUC_s$ is much lower than the $AUC_a$ or $AUC_v$. No statistically significant difference was detected between the $AUC_s$ values of Group A versus Group B, or of Group B versus Group C (Mann-Whitney).

**Figure 2** Mean concentrations of melphalan in perfusate during the hour of ILP —▲—; and in the systemic circulation during and after ILP —□—. (Error bars omitted where s.e.m. <symbol size).

*Pharmacokinetics and Phase I study*

Figure 3 shows the curves describing arterial and venous melphalan levels during isolated limb perfusion in Groups A and B. The mean arterial and venous melphalan concentration time curves for Group A and Group B can be fitted to lines described by bi-exponential equations of the form:

$$C_t = A.e^{-\alpha.t} + B.e^{-\beta.t}$$

where $C_t$ is concentration at time 't' minutes,
A and B are intercepts on the log concentration axis at t = 0 min, of the two linear components of the curve describing logC against time,
and $\alpha$ and $\beta$ are the rate constants of these two components (Bowman & Rand, 1980).

The values for the parameters A, B, $\alpha$ and $\beta$, are given in Table II, along with the half-life ($t_{½}$) values derived from each individual concentration time curve.

Lines were also fitted to describe melphalan concentrations in perfusate during 'mock' perfusion at 37°C and 39°C (Figure 4) according to the mono-exponential equations:

$C_t = 98.4 \times e^{-0.013.t}$ for the 37°C experiment, where $t_{½} = 51.6$ min; and $C_t = 104 \times e^{-0.02}$ for the 39°C experiment, where $t_{½} = 36.6$ min.

It can be seen that the half-life of melphalan in the $\beta$ phase $t_{½}\beta$ of clinical perfusions is longer than the $t_{½}\alpha$ and it approximates to the values for the half-life times in the 'mock' perfusions. Table I and Figure 3 show that increasing the dose of melphalan from 1.5 to 1.75 $mg\, kg^{-1}$ body weight resulted in higher levels of melphalan in perfusate. Comparing drug exposure in perfusate (Table I) the values for $AUC_a$ and $AUC_v$ are both significantly greater in Group B than in Group A ($P$ values $<0.01$, Mann-Whitney). The ratio $AUV_a/AUC_s$ for groups B and C combined was significantly greater than for Group A ($P<0.05$, Mann-Whitney).

The regional toxicity for patients in the phase I study is summarised in Table III according to a simple clinical grading system—from grade I, no subjective or objective evidence of reaction to grade V, a reaction which may require amputation (Wieberdink *et al.*, 1982). In group B we saw Wieberdink grade III reactions on two occasions (considerable erythema and/or oedema with some blistering and slightly disturbed mobility). Since we had not seen such toxicity in either group A or in a previous pilot study of 26 patients it was decided that 1.75 $mg\, kg^{-1}$ body weight should be the maximal dose for our system.

*Bolus or divided dose?*

Figure 5 shows the curves describing melphalan concentration after bolus (Group B) or divided dose (Group C)

**Table I** Melphalan pharmacokinetics in isolated limb perfusion: Dose of melphalan, perfusate $AUC_a$ and $AUC_v$ data for individual patients in Groups A, B and C compared with the $AUC_{0-\infty}$ of systemic concentrations of melphalan ($AUC_s$)

| | Dose (mg) | $AUC_a$ | $AUC_v$ | $AUC_s$ | $AUC_a/AUC_s$ |
|---|---|---|---|---|---|
| *Group A* | 100 | 597 | 504 | 26.5 | 22.5 |
| (1.5 mg kg$^{-1}$ bolus) | 75 | 780 | 932 | 48.9 | 15.95 |
| | 100 | 919 | 848 | 37.5 | 24.5 |
| | 125 | 1140 | 957 | 30 | 38 |
| | 150 | 806 | 748 | 246 | 3.28 |
| | 70 | 1011 | 579 | 287 | 3.52 |
| | 75 | 858 | 869 | 402 | 2.13 |
| | 100 | 1324 | 1360 | 92.3 | 14.34 |
| | 125 | 1229 | 1187 | 98.2 | 12.5 |
| | 130 | 1158 | 1087 | 9.5 | 121.9 |
| | 100 | 1508 | 1105 | 29.4 | 51.3 |
| *Median* | 100 | 1011 | 932 | 48.9 | 15.95 |
| *Group B* | 90 | 1204 | 984 | 361 | 3.33 |
| (1.75 mg kg$^{-1}$ bolus) | 165 | 1912[a] | 1680 | 231 | 8.27 |
| | 140 | 1649[a] | 2083 | 26.4 | 62.5 |
| | 70 | 1144 | 900 | 9.5 | 120.42 |
| | 135 | 4011 | 3056 | 74.3 | 54 |
| | 140 | 2720 | 2030 | 70.1 | 38.8 |
| | 105 | 1758 | 1570 | 34.6 | 50.8 |
| | 90 | 1019 | 963 | 27.2 | 37.5 |
| | 120 | 1275 | 1425 | 24.9 | 51.2 |
| *Median* | 120 | 1649 | 1570 | 34.6 | 50.8 |
| *Group C* | 175 | 2116[b] | 1903 | 62.8 | 33.7 |
| (1.75 mg kg$^{-1}$ divided dose) | 84 | 1443[a] | 1326 | 56.4 | 25.5 |
| | 100 | 1129 | 1018 | 8.6 | 131 |
| | 100 | 988 | 902 | 9.7 | 101.8 |
| | 120 | 2128 | 2024 | 25.2 | 84.4 |
| | 89 | 2180 | 1950 | 35.7 | 61 |
| *Median* | 100 | 1779 | 1614 | 30.5 | 72.7 |

The unit for AUC is the μg min ml$^{-1}$. Toxicity: [a]Grade III; [b]Grade IV (Wieberdink *et al.*, 1982).

**Figure 3** Mean concentrations of melphalan in the perfusate of Group A: --△-- A arterial; --□-- A venous; and Group B: —▲— B arterial; —■— B venous.

**Figure 4** Lines of best-fit describing concentrations of melphalan during 'mock' ILP at 37°C —▲—; and at 39°C · · ·O· · ·.

**Table II a** Summary of the values obtained for the best-fit lines describing concentration time curves for melphalan in arterial and venous perfusate in Groups A and B

| | Parameter | | | |
|---|---|---|---|---|
| | *A* | α | *B* | β |
| *Group A* | | | | |
| Arterial | 48.9 | 0.1 | 16.8 | 0.01 |
| Venous | 42.8 | 0.09 | 13.7 | 0.01 |
| *Group B* | | | | |
| Arterial | 86.5 | 0.09 | 21.4 | 0.003 |
| Venous | 66.5 | 0.19 | 44.3 | 0.02 |

**Table II b** Values for half-life of alpha and beta phases of the fitted lines ($t_{½}α$ and $t_{½}β$) for arterial and venous perfusate in Groups A and B

| | $t_{½}α$ (min) | s.e.m. | $t_{½}β$ (min) | s.e.m. |
|---|---|---|---|---|
| *Group A* | | | | |
| Arterial | 12.27 | 1.06 | 54.45 | 8.64 |
| Venous | 13 | 0.813 | 33.4 | 3.25 |
| *Group B* | | | | |
| Arterial | 13.8 | 0.76 | 57.17 | 11.47 |
| Venous | 17.33 | 2.64 | 44 | 4.91 |

administration of melphalan 1.75 mg kg$^{-1}$ body weight. From data in Table I, there is no significant difference between Group B and Group C (Mann-Whitney) in either the $AUC_a$, $AUC_v$ or $AUC_s$. Table III shows that the regional toxicity was also similar in Groups B and C.

*Calculations of tissue uptake of melphalan*

The results for the calculation of $MEL_{ex}$, according to the Fick principle, in the three groups of patients are given in

**Table III** Regional toxicity in patients treated by isolated limb perfusion in pharmacokinetics studies

| Wieberdink toxicity grade[a] | Group A (1.5 mg kg$^{-1}$) | No. patients Group B (1.75 mg kg$^{-1}$ bolus) | Group C (1.75 mg kg$^{-1}$ divided dose) |
|---|---|---|---|
| I | 1 | 0 | 0 |
| II | 10 | 7 | 4 |
| III | 0 | 2 | 1 |
| IV | 0 | 0 | 1 |
| V | 0 | 0 | 0 |

[a]Wieberdink *et al.*, 1982.

**Figure 5** Mean concentrations of melphalan after a dose of 1.75 mg kg$^{-1}$ given as a bolus to Groups B —▲— B arterial; —■— B venous; and in divided dose to Group C - -△- - C arterial; - -□- - C venous.

Table IV, along with the same calculation for a 'mock' perfusion at 39°C. The $t_{½}$ of melphalan in human plasma *in vitro* was 114 min at 37°C and 60 min at 42°C. In the 'mock' perfusions the $t_{½}$ values were less than *in vitro* incubations, suggesting that hydrolysis proceeds more rapidly or that a significant amount of melphalan may be 'lost' to the constituents of the circuit and to the cellular components of the perfusate. Assuming that hydrolysis is similar in the three clinical groups, the combined results of the calculations in Table IV suggest that approximately 25–40% of administered melphalan distributes to the tissues of the leg (after correction for the proportion 'lost' to the circuit) during isolated limb perfusion.

The slopes of the concentration time curves after bolus dose administration (Figures 3 and 5, Table II) indicate that melphalan disappears from perfusate more rapidly in the first half-hour of perfusion.

**Table IV** Melphalan pharmacokinetics in isolated limb perfusion: Calculated uptake of melphalan by tissues of the leg according to Formula (2): $MEL_{ex} = (AUC_a\text{-}AUC_v) \times Q$

Group A ($n = 11$)

$MEL_{ex}$ = (1030–925) × 443 = 46.5 mg = 44% of mean dose[a]

Group B ($n = 9$)

$MEL_{ex}$ = (1855–1633 × 316 = 70 mg = 60% of mean dose[a]

Group C ($n = 6$)

$MEL_{ex}$ = (1664–1520) × 331 = 47.6 mg = 43% of mean dose[a]

In the clinical groups, mean values for AUC and flow are used. [a]Each of these values appears to be an overestimate, unless one proposes that there is very avid uptake of melphalan by the tissues of the leg. Hence the same calculation is applied to the 39°C mock perfusion data:

$MEL_{ex}$ = (3761–3705) × 400 = 22 mg = 22% of dose, which is the proportion 'lost' to the cellular components of perfusate and to the perfusion circuit.

### Discussion

### *Regional and systemic exposure to melphalan*

There have been a few studies of drug levels achieved in perfusate during ILP with melphalan (Benckhuijsen *et al.*, 1986; Hafstrom *et al.*, 1984; Tonak, 1981; Briele *et al.*, 1985; Minor *et al.*, 1985) but there is little data on systemic levels of melphalan during ILP (Hafstrom *et al.*, 1984; Minor *et al.*, 1985), and the total systemic exposure to melphalan has not been quantified. In our study the peak concentrations of melphalan were much higher in perfusate than in the systemic circulation in all three groups (ratios of peak perfusate: systemic levels in μg ml$^{-1}$ - A 45:0.76, B75.3:0.53, C 44:0.4).

Studies describing the pharmacokinetics of high-dose systemic intravenous melphalan (assay by HPLC) have shown mean AUC values of approximately 400 μg min ml$^{-1}$ in adults (Stotter *et al.*, 1987; Ardiet *et al.*, 1986; Gouyette *et al.*, 1986). From Table I it can be seen that in all our patients the $AUC_a$ and $AUC_v$ are both much higher than this.

We have measured systemic melphalan levels during and for several hours after perfusion to determine the total systemic exposure (Table I), described by the systemic $AUC_{0-\infty}$ ($AUC_s$). In the great majority the $AUC_s$ is much lower, being over 300 μg min ml$^{-1}$ in only two patients. The ratio of $AUC_a/AUC_s$ gives a value which describes the pharmacokinetic advantage achieved by ILP. Although there was a wide range of $AUC_a/AUC_s$, the greater values achieved with the larger dose suggest that we should use 1.75 mg kg$^{-1}$ rather than 1.5 mg kg$^{-1}$.

### *Systemic exposure to melphalan—leak and washout*

Good surgical technique, including awareness of anatomical variations and thorough dissection of the vessels, helps to prevent sudden major 'leaks' to the systemic circulation during perfusion. Even with perfect operative technique, however, there is a variable but inevitable escape from the 'isolated' limb and this may be due to 'leakage' through intra-osseous femoral vessels, vessels passing through the obturator foramen or calcified branches of the cruciate anastomosis which may not be occluded by the tourniquet. After ILP a proportion of the melphalan taken up by the tissues of the limb may diffuse back into bloodstream and be 'washed out' into the systemic circulation.

Studies have shown, using radio-labelled albumin, that the measured 'leak' from perfusate to the systemic circulation during ILP may be as much as 40% (Lejeune & Ghanem, 1987; Hafstrom *et al.*, 1984; Briele *et al.*, 1985), despite very low systemic melphalan levels during perfusion (Hafstrom *et al.*, 1984; Lejeune & Ghanem, 1987). On such evidence it is claimed that the radio-labelled albumin method simply overestimates the 'leak' of melphalan (Lejeune & Ghanem, 1987) but this line of argument neglects several important factors. Albumin is a relatively stable large molecule which will tend to remain within the vascular compartment. In contrast melphalan is continually degraded by hydrolysis in aqueous environments, and the fraction of melphalan which 'leaks' from the isolated limb to the systemic circulation will partition within a much larger volume of distribution (intravascular and interstitial fluid). Furthermore some of the melphalan taken up by the tissues (of the perfused leg and the rest of the body) may be protected from hydrolysis by associating with tissue proteins (Chang *et al.*, 1978*a*; Ehrsson & Lonroth, 1982) and released later, when the melphalan concentration gradients are reversed after perfusion. Thus a significant fraction of the administered melphalan dose could 'leak' and be 'washed out' from the perfusate to systemic circulation, yet produce only low systemic plasma concentrations.

### *Pharmacokinetics of bolus administration*

The concentration time curves describing melphalan in perfusate after bolus administration (Figure 3) are biphasic, conforming to a two compartment model. In groups A and B the $t_{\frac{1}{2}}$ (half-life) for the first, or $\alpha$ phase, was shorter than in the $\beta$ phase. The concentration time curves for 'mock' perfusions were monophasic (Figure 4), conforming to a one compartment model. The $t_{\frac{1}{2}}$ in the $\beta$ phase of clinical perfusion is similar to the half-life observed throughout 'mock' perfusions, when the rate of decay depends on hydrolysis plus losses in the perfusion circuit. It is inferred that the rapid loss of melphalan from perfusate in the $\alpha$-phase of clinical ILP is mainly due to uptake by the tissues of the limb.

*Phase I study and dosimetry*

To take full advantage of the potential benefit of ILP it is important that the tumour-bearing limb is subjected to the maximal safe melphalan exposure, which is a function of time and concentration and which can be quantified by the perfusate AUC. The dose of melphalan used in isolated limb varies from centre to centre. In most series the dose is calculated on the basis of body weight, and the most commonly recommended dose for an iliac perfusion is 1–1.5 mg per kilogram of body weight (Krementz, 1986; Shingleton *et al.*, 1975; Bulman & Jamieson, 1980; Storm & Morton, 1985; Ghussen *et al.*, 1984; Schraffordt Koops *et al.*, 1981). In a few studies it has been suggested that doses as high as 2 mg per kilogram of body weight can be given (Rosin & Westbury, 1980; Fontaine & Jamieson, 1974).

There is a trend towards melphalan dosimetry for ILP according to limb volume (Krementz, 1986). However the measurement of limb volume by water displacement (Wieberdink *et al.*, 1982) can be a cumbersome procedure, particularly in older patients. There have been no reports describing the accuracy and reproducibility of limb volumetry by water displacement. In a small prospective study, it was found (Van Os *et al.*, 1985) when using 10 mg $l^{-1}$ of limb volume that the equivalent of 1.77 mg $kg^{-1}$ body weight could be given safely in their patients (their previous mean dose 1.44 mg $kg^{-1}$). Similar results would have been obtained in their study if the higher doses had simply been administered on the basis of body weight.

For patients of 'average build' body weight is an acceptable basis on which to calculate dose in ILP, but the use of limb volume dosimetry may be of specific value in the management of patients who have an abnormal habitus, or amputees.

It is generally accepted that a lower total dose of melphalan should be used when perfusing smaller regions of the body e.g. axillary perfusion. In this setting the limb volume method for dosimetry results in doses which are probably inadequate (Van Os *et al.*, 1985) since they are even lower than those calculated on the basis of body weight (Krementz *et al.*, 1985). Although the rationale is plausible the practical benefits of routine dosimetry by limb volume are unclear.

It has been recommended that other factors to be considered when calculating the dose of melphalan to be administered include complexion and hair colour (McBride & Clark, 1971; Schraffordt Koops *et al.*, 1977)—fair-skinned red-heads being supposedly more susceptible to toxicity than those of a dark complexion but these recommendations have not been validated.

Body weight is obtained easily and reproducibly on the ward, without special equipment. We believed that since no formal phase I study had been carried out using body weight for dosimetry such a study should be done.

In the phase I study it was found that the dose of melphalan could be increased form 1.5 mg $kg^{-1}$ body weight (Group A) to 1.75 mg $kg^{-1}$ (Group B) with an acceptable slight increase in regional toxicity (Table III). The peak levels of melphalan were higher after 1.75 mg $kg^{-1}$ than after 1.5 mg $kg^{-1}$, and the higher levels were maintained during perfusion. Comparison of the perfusate AUC data for Groups A and B (Table I) confirms that the higher dose resulted in significantly greater bio-availability of melphalan within the limb, without any increased systemic exposure. In no patient was a significant leak suspected but there was a wide range in $AUC_s$ (8.6–402 μg min $ml^{-1}$). None of the five cases which had an $AUC_s$ value greater than 200 μg min $ml^{-1}$ suffered significant systemic toxicity and, in particular, there was no detected bone marrow toxicity. This may be due to the relatively slow release of melphalan from the tissues of the perfused limb, which will be less likely to produce high peak systemic levels and 'saturate' the bone marrow than a single large intravenous bolus as given during high dose systemic melphalan therapy.

In reports of ILP it is remarkable that the volume of perfusate, which critically determines the effective concentration of melphalan, has been so neglected. In fact, the volume of prime is often omitted in reports (Storm & Morton, 1985; Schraffordt Keeps *et al.*, 1987; Sugarbaker & McBride, 1976; Mikhail *et al.*, 1984; Rege *et al.*, 1983), even in studies of melphalan pharmacokinetics (Hafstrom *et al.*, 1984; Osterheld *et al.*, 1988).

Formerly it was believed that the perfusate volume was mainly determined by the prime (Wieberdink *et al.*, 1982), but the total volume of perfusate also includes blood trapped in the limb vessels at the start of perfusion. The volume of prime is easily measured directly but the volume of blood trapped in the limb vasculature is probably more variable. The volume of priming fluid varies in descriptions of ILP according to different authors from 600 ml (Krementz *et al.*, 1987; Hansson *et al.*, 1977) to two litres (Rosin & Westbury, 1980). We used a fixed volume of prime and a standard cannulation sequence, but some of the variability in our pharmacokinetic results is likely to be due to the variable volume of trapped limb blood which contributes to the total perfusate volume.

Elegant methods have recently been described for estimating perfusate volume (Benckhuijsen *et al.*, 1986; Lejeune & Ghanem, 1987). The validity of these methods has not been confirmed, but the attraction is that, based on pharmacokinetic parameters, accurate knowledge of the perfusate volume would allow the administration of a dose calculated to produce a predictable concentration (Siddik *et al.*, 1989) in perfusate. Assuming that cytotoxic effect and toxicity are mainly dependent on melphalan concentration and AUC, this would represent an advance on dosimetry by body weight or limb volume. In an ideal situation, this new approach would be combined with knowledge of the relative sensitivities of an individual patient's tumour (and normal tissue) to cytotoxic drugs, allowing 'tailored' therapy.

*Bolus of divided dose?*

The original rationale for dividing the dose was to minimise the consequences of 'leakage' and it is not clear whether there is any pharmacokinetic advantage in bolus or divided dose administration.

Once we determined that melphalan could be safely given as a bolus dose of 1.75 mg $kg^{-1}$ body weight (Group B, Table III), we set out to discover whether divided dose administration at this dose level (Group C) resulted in greater $AUC_a$ and $AUC_v$ values. There was no significant difference in these values between the two groups. However one patient in Group C (total dose 175 mg; $AUC_a$ 2116 min μg $ml^{-1}$) suffered a severe reaction (Wieberdink Grade IV).

We have shown that up to 165 mg of melphalan can be given safely as a bolus (calculated as 1.75 mg $kg^{-1}$ body weight) during ILP, and that there is no apparent pharmacokinetic advantage in divided dose administration.

*Calculations of tissue uptake of melphalan*

It would clearly be desirable to know what proportion of administered melphalan is taken up by the tissues of the leg, and also to discover how that proportion is distributed among the different tissues (especially any differences between benign and malignant). With this knowledge the effects of various manipulations (e.g. changing dose of drug, duration of perfusion or temperature) could be assessed. Attempts have been made to estimate the proportion of administered melphalan which distributes to the tissues of the leg semi-quantitatively (Briele *et al.*, 1985) and quantitatively (Benckhuijsen *et al.*, 1988). One previous publication describes the measurement of tissue concentrations of melphalan achieved by ILP (Stotter *et al.*, 1987) in three patients. It has been suggested that cellular uptake mechanisms for melphalan may be saturable in the perfused limb (Benckhuijsen *et al.*, 1986; Briele *et al.*, 1985).

This is the first study where paired arterial and venous samples of perfusate were obtained for melphalan analysis throughout the hour of ILP. Hence we could use the perfusate AUC data in Formula (2) which is derived from the Fick principle. Uncorrected, the results tend to overestimate the amount of melphalan taken up by the leg ($MEL_{ex}$) and 45–60% of administered dose. It is important to realise that the melphalan concentration measured is the plasma concentration, and that although hydrolysis is corrected for, melphalan is known to rapidly associate with the cellular components of blood (Briele *et al.*, 1985; Greig *et al.*, 1987 in a ratio of cells:plasma equal to approximately 1:1 (Benckhuijsen *et al.*, 1986). During the hour of perfusion a fraction of the administered dose (approximating to the haematocrit as %) partitions to the red blood cells, and this accounts for the 22% correction factor as calculated by applying Formula (2) to the 'mock' perfusion (see Table IV).

Dividing the dose (Group C) may allow continued drug uptake throughout the period of perfusion but it is not clear whether this is advantageous, or whether it may be preferable to generate early high peaks by bolus administration. Yet another strategy would be to administer the cytotoxic by infusion into the arterial line of the circuit, while slowing the flow rate to maximise the effect of first-pass extraction. Homogeneous mixing of melphalan within the perfusate might be achieved by injecting the drug via the arterial line during the course of one circulation time (Wieberdink *et al.*, 1982).

Estimates of tissue uptake based on changes in perfusate concentration of melphalan (Briele *et al.*, 1985; Benckhuijsen *et al.*, 1988), including our Fick based calculations, are merely indicators of the dynamic situation in the limb as a whole, but the critical question relates to the concentration of melphalan which is achieved at the site of action i.e. tumour cell DNA. The changes in perfusate concentration of melphalan will be, at best, crude indicators of critical events which govern the passage of melphalan from the capillary lumen to the melanoma cell nucleus. The duration of ILP with melphalan is usually one hour (Krementz *et al.*, 1987; Martijn *et al.*, 1986; Benckhuijsen *et al.*, 1986) but it may be as short as 45 min (Mikhail *et al.*, 1984) or as long as two and a half hours (Stehlin, 1969; Hafstrom *et al.*, 1984).

There is no clinical trial based evidence to suggest that the optimal duration for ILP has been found. In spite of the short half-life of melphalan in aqueous solution, and the relatively short time of exposure involved in ILP, it may be that the drug is somewhat protected from hydrolysis once it leaves the vascular compartment. Furthermore, the drug in the tissues may continue to form cross-links for hours after exposure (Hansson *et al.*, 1987). Hence there may be relatively little therapeutic gain achievable by prolonged clinical ILP, if maximal safe doses are already being given. We believe that it would be premature to advocate shorter periods of perfusion.

### Conclusions

Isolated limb perfusion successfully and consistently achieves the aim of exposing the tumour-bearing limb to high concentrations of melphalan, while minimising systemic exposure.

Following bolus administration of melphalan the concentration time curve is biphasic, with a mean $t_{\frac{1}{2}}\alpha$ of 12.27–17.33 min, and mean $t_{\frac{1}{2}}\beta$ of 33.4–57.17 min. Melphalan seems to be taken up by the tissues of the leg mainly during the first 30 min of isolated limb perfusion. However perfusion should last longer than 30 min to maintain the concentration gradients which drive the drug through diffusion barriers to the target cells.

In a phase I study we have shown that, using our standard technique of isolated limb perfusion, melphalan can be given safely in a bolus dose of 1.75 mg $kg^{-1}$ body weight (up to 165 mg total dose). This higher dose yielded a significantly greater value of $AUC_a/AUC_s$ than did 1.5 mg $kg^{-1}$.

There was no pharmacokinetic advantage in divided dose administration, and no increased regional toxicity as a consequence of the high peak levels which occur during bolus dose administration.

R.N.S. was supported by a grant from the Cancer Research Campaign (SP1837).

### References

ARDIET, C., TRANCHAND, B., BIRON, P., REBATTU, P. & PHILIP, T. (1986). Pharmacokinetics of high-dose intravenous melphalan in children and adults with forced diuresis. Report in 26 patients. *Cancer Chemother. Pharmacol.*, **16**, 300–305.

BENCKHUIJSEN, C., KROON, B.B.R., VAN GEEL, A.N. & WIEBERDINK, J. (1988). Regional perfusion treatment with melphalan for melanoma in a limb: An evaluation of drug kinetics. *Eur. J. Surg. Oncol.*, **14**, 157–163.

BENCKHUIJSEN, C., VAROSSIEAU, F.J., HART, A.A.M., WIEBERDINK, J. & NOORDHOEK, J. (1986). Pharmacokinetics of melphalan in isolated limb perfusion of the limbs. *J. Pharmacol. Exp. Ther.*, **237**, 583–588.

BOWMAN, W.C. & RAND, M.J. (1980). Absorption, distribution, excretion and metabolism of drugs. Biopharmaceutics and pharmacokinetics. In *Textbook of Pharmacology*, Bowman, W.C. & Rand, M.J. (eds), p. 40.57–8. Blackwell: Oxford.

BRIELE, H.A., DJURIC, M., JUNG, D.T., MORTELL, T., PATEL, M.K. & DAS GUPTA, T.K. (1985). Pharmacokinetics of melphalan in clinical isolation perfusion of the extremities. *Cancer Res.*, **45**, 1885–1889.

BULMAN, A.S. & JAMIESON, C.W. (1980). Isolated limb perfusion with melphalan in the treatment of malignant melanoma. *Br. J. Surg.*, **67**, 660–662.

CHANG, S.Y., ALBERTS, D.S., FARQUHAR, D., MELNICK, L.R., WALSON, P.D. & SALMON, S.E. (1978*a*). Hydrolysis and protein binding of melphalan. *J. Pharm. Sci.*, **67**, 682–684.

CHANG, S.Y., ALBERTS, D.S., MELNICK, L.R., WALSON, P.D. & SALMON, S.E. (1978*b*). High pressure liquid chromatographic analysis of melphalan in plasma. *J. Pharm. Sci.*, **67**, 679–682.

EHRSSON, H. & LONROTH, U. (1982). Degradation of melphalan in aqueous solutions–influence of human albumin binding. *J. Pharm. Sci.*, **71**, 826–827.

FONTAINE, C.J. & JAMIESON, C.W. (1974). Perfusion in limb melanoma: indications and results. *Proc. Roy. Soc. Med.*, **67**, 99–100.

GANONG, W.F. (1981). The heart as a pump: Cardiac output. In *Review of Medical Physiology. 10th ed.* Ganong, W.F. (ed.) p. 445. Lange: Los Altos, California.

GHUSSEN, F., NAGEL, K., GROTH, W., MULLER, J.M. & STUTZER, H. (1984). A prospective randomised study of regional extremity perfusion in patients with malignant melanoma. *Ann. Surg.*, **200**, 764–768.

GOODMAN, A.G., GILMAN, L.S., RALL, T.W. & MURAD, F. (1985). *The Pharmacological Basis of Therapeutics (7th ed.)*. p. 1. MacMillan: New York.

GOUYETTE, A., HARTMANN, O. & PICO, J.-L. (1986). Pharmacokinetics of high-dose melphalan in children and adults. *Cancer Chemother. Pharmacol.*, **16**, 184–189.

GREIG, N.S., SWEENEY, D.J. & RAPOPORT, S.I. (1987). Melphalan concentration dependent plasma protein binding in healthy humans and rats. *Eur. J. Clin. Pharmacol.*, **32**, 179–185.

HAFSTROM, L., HUGANDER, A., JONSSON, P.-E., WESTLING, H. & EHRSSON, H. (1984). Blood leakage and melphalan leakage from the perfusion circuit during regional hyperthermic perfusion for malignant melanoma. *Cancer Treat. Rep.*, **68**, 867-872.

HANSSON, J., LEWENSOHN, R., RINGBORG, U. & NILSSON, B. (1987). Formation and removal of DNA cross-links induced by melphalan and nitrogen mustard in relation to drug-induced cytotoxicity in human melanoma cells. *Cancer Res.*, **47**, 2631-2637.

HANSSON, J.A., SIMERT, G. & VANG, J. (1977). The effect of regional perfusion treatment on recurrent melanoma of the extremities. *Acta. Chir. Scand.*, **143**, 33-39.

KREMENTZ, E.T. (1986). Lucy Wortham James lecture: Regional perfusion. Current sophistication, what next? *Cancer*, **57**, 416-432.

KREMENTZ, E.T., CARTER, R.D., SUTHERLAND, C.M. & MUCHMORE, J.H. (1987). Chemotherapy by regional perfusion for limb melanoma. *Am. Surg.*, **53**, 133-140.

KREMENTZ, E.T., RYAN, R.F., CARTER, R.D., SUTHERLAND, C.M. & REED, R.J. (1985). Hyperthermic regional perfusion for melanoma of the limbs. In *Cutaneous melanoma. Clinical management and treatment results world-wide*, Balch, C.M. & Milton, G.W. (eds) p. 171-190. J.P. Lippincott: Philadelphia.

LEJEUNE, F.J. & GHANEM, G.E. (1987). A simple and accurate new method for cytostatics dosimetry in isolation perfusion of the limbs based on exchangeable blood volume determination. *Cancer Res.*, **47**, 639-643.

MCBRIDE, C.M. & CLARK, R.L. (1971). Experience with L-phenylalanine mustard dihydrochloride in isolation-perfusion of extremities for malignant melanoma. *Cancer*, **28**, 1293-1296.

MARQUARDT, D.E. (1963). An algorithm for least-squares estimation of nonlinear parameters. *J. Soc. Ind. Appl. Math.*, **11**, 431-441.

MARTIJN, H., SCHRAFFORDT KOOPS, H., MILTON, G.W. & 4 others (1986). Comparison of two methods of treating primary malignant melanomas Clark level IV and V, thickness 1.5 mm and greater, localised on the extremities. Wide surgical excision with and without adjuvant regional perfusion. *Cancer*, **57**, 1923-1930.

MIKHAIL, R.A., BODDIE, A.W., AMES, F.C., ZIMMERMAN, S.O. & MCBRIDE, C.M. (1984). Effect of variation of drug dosage on disease control and regional toxicity in phrophylactic perfusion for stage I extremity melanoma. *J. Surg. Oncol.*, **27**, 215-218.

MINOR, D.R., ALLEN, R.E., ALBERTS, D., PENG, Y., TARDELLI, G. & HUTCHINSON, J. (1985). A clinical and pharmacokinetic study of isolated limb perfusion with heat and melphalan for melanoma. *Cancer*, **55**, 2638-2644.

OSTERHELD, H.K.O., MUSCH, E., VON UNRUH, G.E., LOOS, U., RAUSCHECKER, H. & MUHLENBRUCH, B.J. (1988). A sensitive high-performance liquid chromatographic assay for melphalan and its hydrolysis products in blood and plasma. *Cancer Chemother. Pharmacol.*, **21**, 156-162.

REGE, V.B., LEONE, L.A., SODERBERG, C.H. & 4 others (1983). Hyperthermic adjuvant perfusion chemotherapy for stage I malignant melanoma of the extremity with literature review. *Cancer*, **52**, 2033-2039.

ROSIN, R.D. & WESTBURY, G. (1980). Isolated limb perfusion for maligant melanoma. *The Practitioner*, **224**, 1031-1036.

SCHRAFFORDT KOOPS, H., BEEKHUIS, H., OLDHOFF, J., OOSTERHUIS, J.W., VAN DER PLOEG, E. & VERMEY, A. (1981). Local recurrence and survival in patients with (Clark level IV/V and over 1.5 mm thickness) stage I malignant melanoma of the extremities after regional perfusion. *Cancer*, **48**, 1952-1957.

SCHRAFFORDT KOOPS, H., OLDHOFF, J., OOSTERHUIS, J.W. & BEEKHUIS, J. (1987). Isolated regional perfusion in malignant melanoma of the extremities. *World J. Surg.*, **11**, 527-533.

SCHRAFFORDT KOOPS, H., OLDHOFF, J., VAN DER PLOEG, E., VERMEY, A. & EIBERGEN, R. (1977). Regional perfusion for recurrent malignant melanoma of the extremities. *Am. J. Surg.*, **133**, 221-224.

SHINGLETON, W.W., SEIGLER, H.F., STOCKS, L.H. & DOWNS, R.W. (1975). Management of recurrent melanoma of the extremity. *Cancer*, **35**, 574-579.

SIDDIK, Z.H., EDWARDS, M.J. & BODDIE, A.W. (1989). Isolated limb perfusion with chemotherapeutic agents for melanoma: a reevaluation of drug dosimetry. *Eur. J. Cancer Clin. Oncol.*, **25**, 1393-1397.

STEHLIN, J.S. (1969). Hyperthermic perfusion with chemotherapy for cancers of the extremities. *Surg. Gyn. Obstet.*, **129**, 305-308.

STORM, F.K. & MORTON, D.L. (1985). Value of therapeutic hyperthermic limb perfusion in advanced recurrent melanoma of the lower extremity. *Am. J. Surg.*, **150**, 32-35.

STOTTER, A.T., ROBINSON, B., CLUTTERBUCK, R. & 4 others (1987). A comparison of melphalan levels achieved in isolated limb perfusion and systemic high dose therapy. *J. Exp. Clin. Cancer Res.*, **6**, 161-165.

SUGARBAKER, E.V. & MCBRIDE, C.M. (1976). Survival and regional disease control after isolation-perfusion for invasive stage I melanoma of the extremities. *Cancer*, **37**, 188-198.

TONAK, J. (1981). Die hypertherme Zytostatikaperfusion beim malignen Melanom: Eine experimentelle und klinische Untersuchung. Experimenteller Teil. In *Aktuelle Onkologie I*, Nagel, G., Sauer, R. & Schreiber, H.W. (eds) p. 15-31. W. Zuckschwerdt Verlag: Munchen.

VAN OS, J., SCHRAFFORDT KOOPS, H. & OLDHOFF, J. (1985). Dosimetry of cytostatics in hyperthermic regional isolated perfusion. *Cancer*, **55**, 698-701.

WIEBERDINK, J., BENCKHUIJSEN, C., BRAAT, R.P., VAN SLOOTEN, E.A. & OLTHUIS, G.A.A. (1982). Dosimetry in isolation perfusion of the limbs by assessment of perfused tissue volume and grading of toxic tissue reactions. *Eur. J. Cancer Clin. Oncol.*, **18**, 905-910.